# APPLYING IMPLEMENTATION SCIENCE FRAMEWORKS TO DELIVER INTERVENTIONS IN THE NURSING HOME SETTING

**DOI:** 10.1093/geroni/igae098.0156

**Published:** 2024-12-31

**Authors:** Katherine Abbott, Kathleen Unroe

**Affiliations:** Chair; Discussant

## Abstract

Approximately 1.2 million people reside in more than 15,000 nursing homes (NH) in the United States. The 2022 NASEM report on NH quality identified persistent issues to the provision of high-quality NH care in the United States. To improve NH quality, implementation science (IS) theories, methods, and frameworks are necessary to close the gap in delays of evidence based therapies and models of care reaching NH residents. This symposium highlights IS work in the NH setting that seeks to improve the quality of care delivered. Our first presentation reports findings on the pre-implementation work of a five-year VA funded study to ensure documentation of life sustaining treatment with over n=50 clinical and administrative staff. The second presentation utilizes the Practical, Robust Implementation and Sustainability Model (PRISM) to identify factors to consider before intervention implementation with results from n=5 focus groups with 27 NH Staff. The third presentation describes the modifications n=23 NH providers made to the Individualized Positive Psychosocial Interaction as well as their motivations behind the modifications via the Framework for Reporting Adaptations and Modifications to Evidence-Based Implementation Strategies. The fourth presentation reports findings from n=6 focus group sessions representing n=23 people living with dementia, their care partners and NH staff using the Readiness Assessment Pragmatic Trial Model to inform what mattered to them when implementing and evaluating a remote format of Me & My Wishes. The Discussant, Dr. Kathleen Unroe, will discuss the implications to advancing IS as well as the applicability of IS in the NH setting. Research in Quality of Care Interest Group Sponsored Symposium

